# Majority of human traits do not show evidence for sex-specific genetic and environmental effects

**DOI:** 10.1038/s41598-017-09249-3

**Published:** 2017-08-17

**Authors:** Sven Stringer, Tinca J. C. Polderman, Danielle Posthuma

**Affiliations:** 1 Department of Complex Trait Genetics, VU University, Center for Neurogenomics and Cognitive Research, Amsterdam Neuroscience, Amsterdam, The Netherlands; 2 Department of Clinical Genetics, VU University Medical Centre, Amsterdam Neuroscience, Amsterdam, The Netherlands

## Abstract

Sex differences in the etiology of human trait variation are a major topic of interest in the social and medical sciences given its far-reaching implications. For example, in genetic research, the presence of sex-specific effects would require sex-stratified analysis, and in clinical practice sex-specific treatments would be warranted. Here, we present a study of 2,335,920 twin pairs, in which we tested sex differences in genetic and environmental contributions to variation in 2,608 reported human traits, clustered in 50 trait categories. Monozygotic and dizygotic male and female twin correlations were used to test whether the amount of genetic and environmental influences was equal between the sexes. By comparing dizygotic opposite sex twin correlations with dizygotic same sex twin correlations we could also test whether sex-specific genetic or environmental factors were involved. We observed for only 3% of all trait categories sex differences in the amount of etiological influences. Sex-specific genetic factors were observed for 25% of trait categories, often involving obviously sex-dependent trait categories such as puberty-related disorders. Our findings show that for most traits the number of sex-specific genetic variants will be small. For those traits where we do report sexual dimorphism, sex-specific approaches may aid in future gene-finding efforts.

## Introduction

In the past decade, several studies have highlighted the importance of differential genetic and environmental effects on a variety of traits across males and females^1–5^. For example, a study performed in 806 subjects from a genetically isolated Hutterite population showed sex differences in X-linked and autosomal additive genetic effects on several anthropometric traits, such as height, fasting insulin, and triglycerides^1^. Similarly, several robust sex-specific genetic effects have been identified outside the sex chromosomes in human^3^ and animal studies^6–8^. In addition, sex-specific environmental effects have been observed in obesity, where boys and girls differ in susceptibility to their social environment^9^. The presence of sex-specific etiological effects in human traits has far-reaching implications. A strong contribution of sex-specific effects in trait variation would for instance imply a difference in etiology between males and females for the same disorder, which would require sex-specific treatments. To assess the overall importance of sex-specific genetic effects across human traits, estimates of sex-specific heritability and male-female genetic correlations are most informative. While heritability quantifies the relative contribution of genetic effects compared to environmental effects in a trait, male-female co-heritability quantifies to what extent the same genetic variants play a role in males and females. Both measures are largely independent and complementary, and can be assessed using pairs of monozygotic (MZ) and dizygotic (DZ) twins, including same-sex and opposite DZ twins.

Twin studies have contributed enormously to our understanding of the relative contribution of genetic and environmental effects across human traits. A recently published meta-analysis of virtually all twin studies^10^ provided sex-specific estimates for the contribution of genes and environment across all traits investigated thus far. However, this study did not include an in-depth analysis of the extent to which male and female heritability estimates differed, nor did it report on the co-heritability in males and females. In the present study we therefore use single-sex and opposite-sex twin correlations to systematically assess the overall contribution of sex-specific genetic effects as well as the male-female genetic overlap across all investigated domains of human traits.

The use of twin correlations allows testing of three sex-specific hypotheses. The first hypothesis is that the difference between the monozygotic twin correlation (r_MZ_) and dizygotic twin correlation (r_DZ_) is similar across males (M) and females (F) ((r_MZM_-r_DZM_) = (r_MZF_-r_DZF_)). This can be interpreted as a test of whether the influence of (additive) genetic effects on the population variance is the same in males and females (hypothesis of equal amount of heritability). The second hypothesis is that the difference between 2*r_DZ_ and r_MZ_ is similar across sexes, thus ((2r_DZM_-r_MZM_) = (2r_DZF_-r_MZF_))^11^. This can be interpreted as a test of whether the relative influence of the shared environment is the same in males and females (hypothesis of equal amount of shared environmental influences) The third hypothesis that can be tested with twin data is that the observed correlation in DZ same-sex twins (r_DZSS_), here defined as the male and female DZ twin correlation midpoint 1/2 r_DZM_ + 1/2 r_DZF_, is the same as the observed correlation in DZ twins of opposite sex (r_DZSS_ = r_DOS_). This is a test of whether the same genes and regulatory regions (and/or shared environmental factors) affect the trait of interest in males and females (hypothesis of full co-heritability).

## Results

To assess the contribution of sex-specific genetic effects we used the MATCH database of twin correlations of Polderman *et al*.^10^, of which we selected all traits for which sex-specific twin correlations and their standard errors (SEs) were available. The three hypotheses as introduced above were tested per ‘trait’ as reported in the original study as well as per ‘trait category’ following the official ICF^12^ and ICD-10^13^ sub-chapter classification (see Methods for details). We report on 50 trait categories based on 2,608 reported traits to test our hypotheses (see Table 1).

**Table 1:** Descriptive statistics of sex-specific twin correlations and their derivatives used in this study.

Null hypothesis tested	N traits	Total N twin-pairs*	N trait categories**
r_MZM_-r_DZM_ = r_MZF_-r_DZF_ (equal heritability)	2,608	2,335,920	50
2r_DZM_-r_MZM_* = *2r_DZF_-r_MZF_ (equal shared environment)	2,608	2,335,920	50
r_DZSS_ = r_DOS_ (full co-heritability)	1,922	1,433,967	36

*Selected for at least 5 pairs per individual study. **With at least 500 pairs included in the meta-analytic estimate, and based on at least 10 studies.

### Sex differences per trait

**Figure 1:**
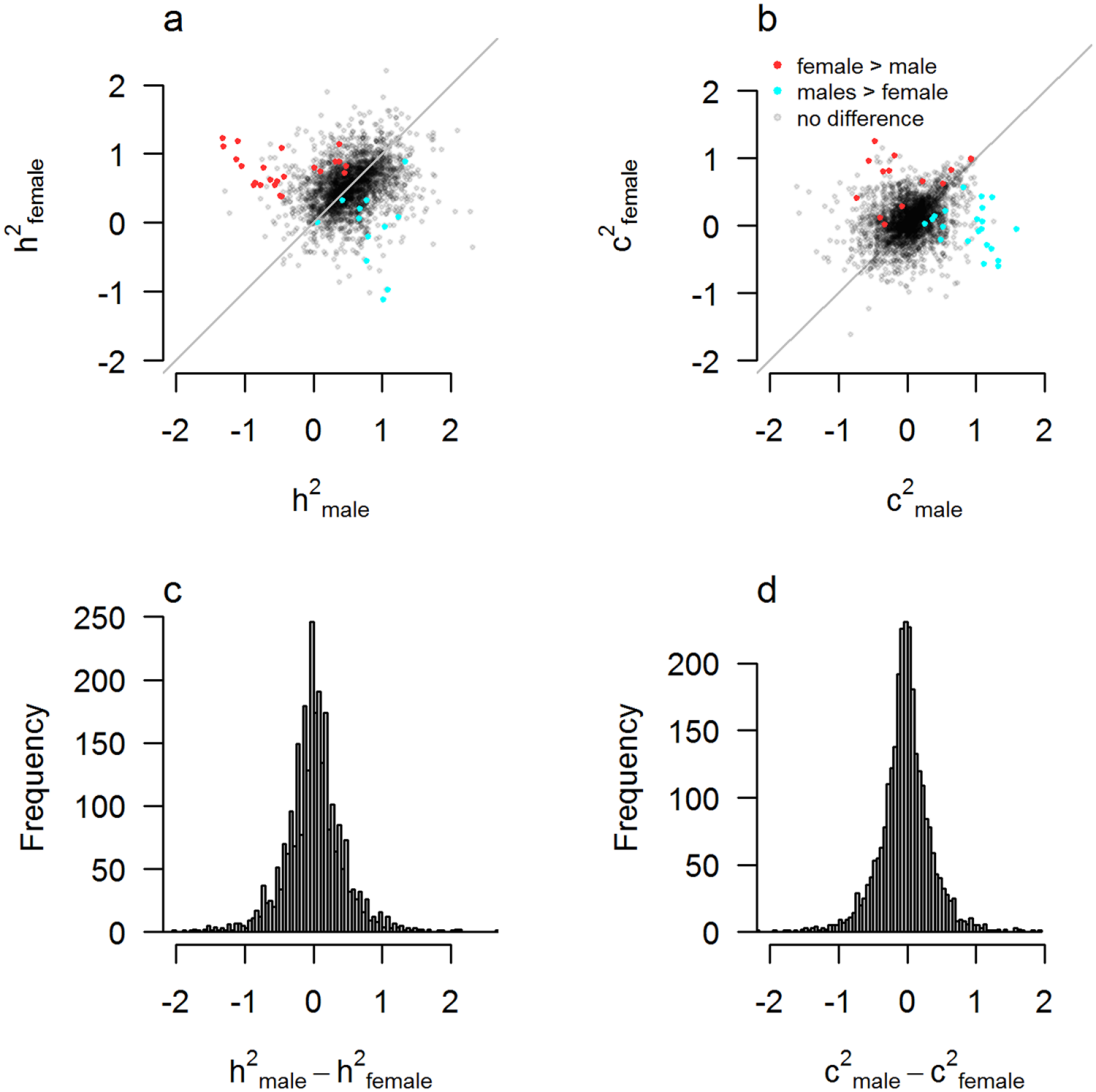
Estimates of genetic and environmental influences for males versus females across individual study traits. Scatterplots of estimates of (**a**) heritability (h^2^ = 2(r_MZ_ − r_DZ_)) and (**b**) amount of shared environment (c^2^ = 2r_DZ_ − r_MZ_) for males versus females. Red dots indicate traits for which the female estimate is significantly larger than the male estimate after. Blue dots indicate traits for which male heritability is significantly larger than female heritability. Grey dots indicate no statistically significant difference after Bonferroni correction for 2,608 trait statistics. The bottom graphs show the distribution of male-female differences in genetic effects (**c**) and shared environmental effects (**d**).

For each trait we tested whether r_MZM_-r_DZM_ = r_MZF_-r_DZF_. Of all 2,608 traits the relative contribution of genetic influences was significantly different between males and females for 1% (37) of the traits after Bonferroni corrected p-value of 1.5 × 10^−5^, while 12% (309) of traits were significant at the uncorrected 0.05 level. The test for equal shared environment variance resulted in a significantly different contribution of the environment between males and females in 1% (35) of traits after correction (p < 1.5 × 10^−5^) and 12% (311) at the 0.05 level of significance (Fig. 1a and b).

Note that although it is impossible to have a negative h^2^ and c^2^ in the population, due to the sometimes large sampling error of the observed twin correlations, sample estimates of h^2^ and c^2^ can be negative. Although it is possible to truncate these negative values to zero, this would result in biased estimates of the sex differences in h^2^ and c^2^, making hypothesis testing less reliable. In other words, a significant sex difference in h^2^ or c^2^ driven by a negative value only reflects that the null hypothesis of no sex difference can be rejected. It does not imply that h^2^ or c^2^ are negative on the population level. In addition, some statistically significant sex-differences are close to zero, due to small standard errors. This illustrates that statistically significant sex-differences are not necessarily large. The size of these significant sex-differences should therefore be taken into account as well.

The distribution of the genetic and environmental differences between males and females was symmetrically distributed around zero with a sharp peak and long tails (Fig. 1c and d). The sex-differences in h^2^ and c^2^ are not normally distributed due to the large variation in standard errors. Drawing test statistics under the null hypothesis of no sex-differences from a normal distribution based on the same variation in standard errors, shows a similarly peaked distribution (see Supplementary Fig. S1).

**Figure 2:**
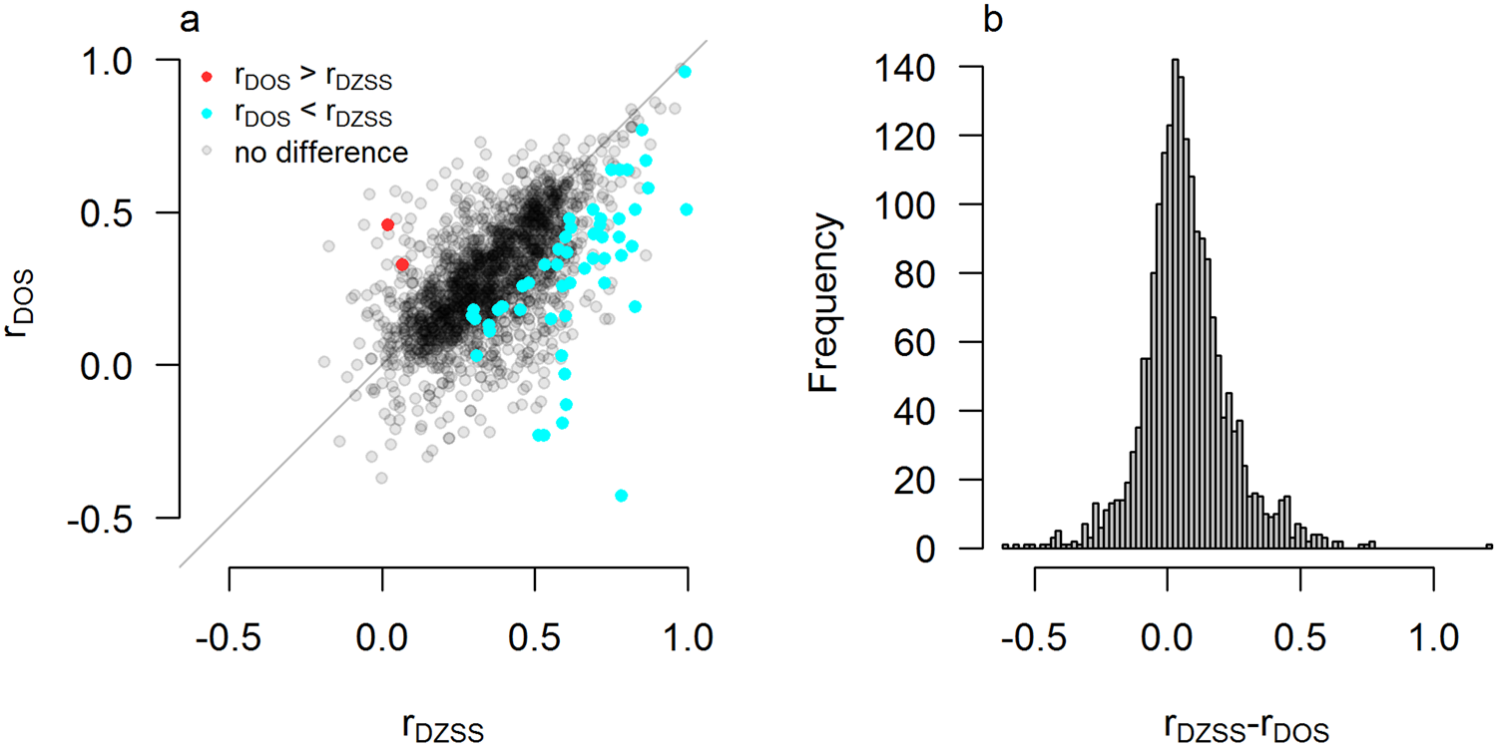
Estimates of co-heritability between males and females across 1,922 individual study traits. (**a**) Scatterplot of all dizygotic (DZ) same-sex midpoint correlations (r_DZSS_ = 1/2 r_DZM_ + 1/2 r_DZF_) versus DZ opposite-sex correlations (r_DOS_). For each study trait it was tested whether r_DZSS_ = r_DOS_. Red dots indicate that r_DOS_ was significantly larger than r_DZSS_. Blue dots indicate that r_DZSS_ was larger than r_DOS_. Grey dots indicate no statistically significant difference after Bonferroni correction for 1,922 individual study traits. (**b**) Distribution of all DZ same-sex midpoint and r_DOS_ differences. Although most differences are close to zero, opposite-sex correlations are on average larger than same-sex DZ twin correlations.

When testing for the contribution of sex-specific effects in these traits (r_DZSS_ = r_DOS_) in 1,922 within-study comparisons, 3% (55) of traits were significantly different from each other at the Bonferroni corrected level of p < 2.6 × 10^−5^, while 23% (447 traits) were significant at an alpha of 0.05 (Fig. 2a). All statistically significant effects involved r_DZSS_ ≥ r_DOS_, which is in the expected direction. This pattern was also reflected in a slightly skewed distribution of the difference between the opposite and same-sex correlation (Fig. 2b). See Figure S1 for the distribution under the symmetric null hypothesis.

### Sex differences per trait category

**Figure 3:**
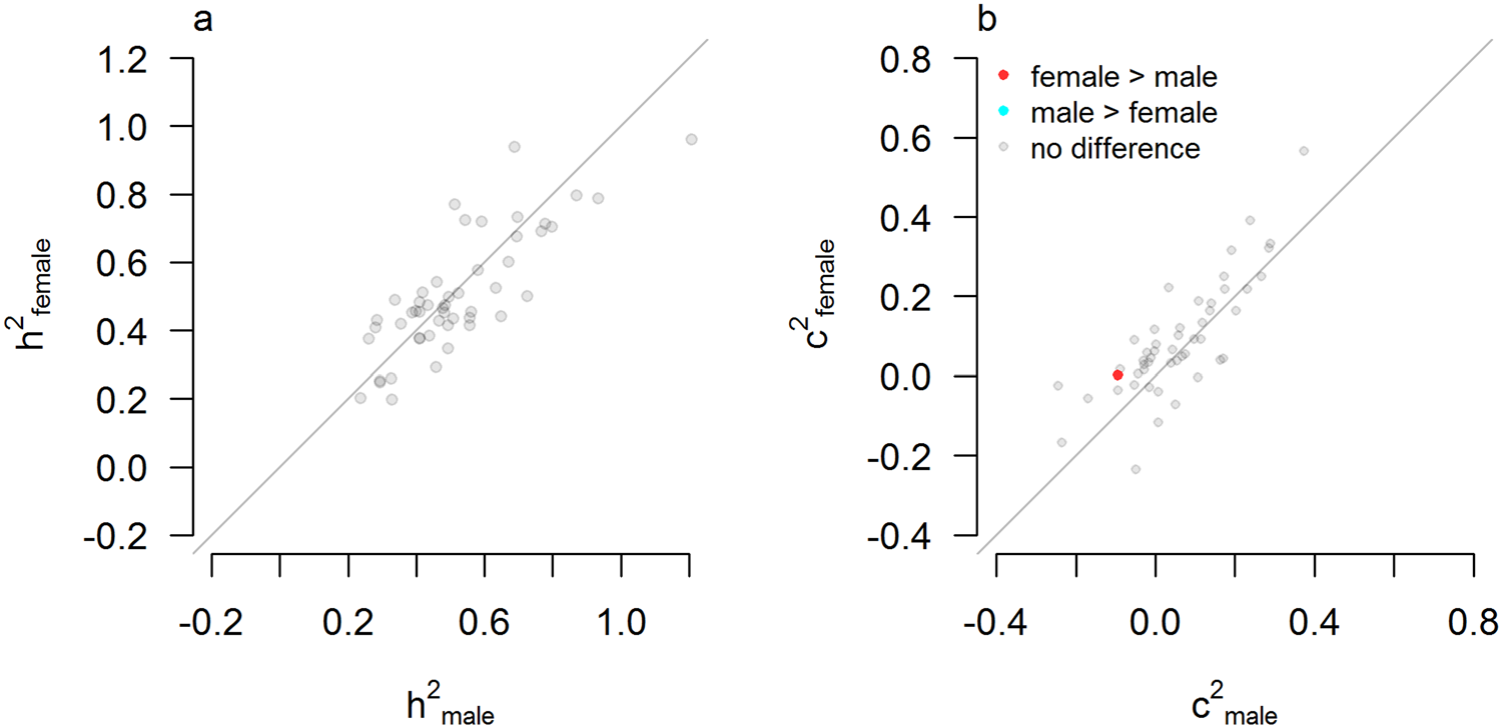
Estimates of genetic and environmental influences for males versus females across 50 trait categories. Scatterplots of estimates of (**a**) heritability (h^2^ = 2(r_MZ_ − r_DZ_)) and (**b**) amount of shared environment (c^2^ = 2r_MZ_ − r_DZ_) for males versus females. Red dots indicate traits for which the female estimate is significantly larger than the male estimate. Blue dots indicate traits for which male heritability is significantly larger than female heritability. Grey dots indicate no statistically significant difference after Bonferroni correction for 50 trait category statistics. Except for *Function of Brain* no trait categories shows robust statistical evidence for sex-specific heritabilities or environmental influences.

Here we tested whether sex differences clustered within trait categories. Testing for heritability differences between males and females in 50 trait categories did not reveal any statistically different effects (Fig. 3a). Only the shared environmental estimate (2r_DZ_-r_MZ_) of the trait category *Function of Brain* was significantly higher for females than for males, although the difference was modest (−0.1) and both male and female estimates were close to zero (Fig. 3b).

**Figure 4:**
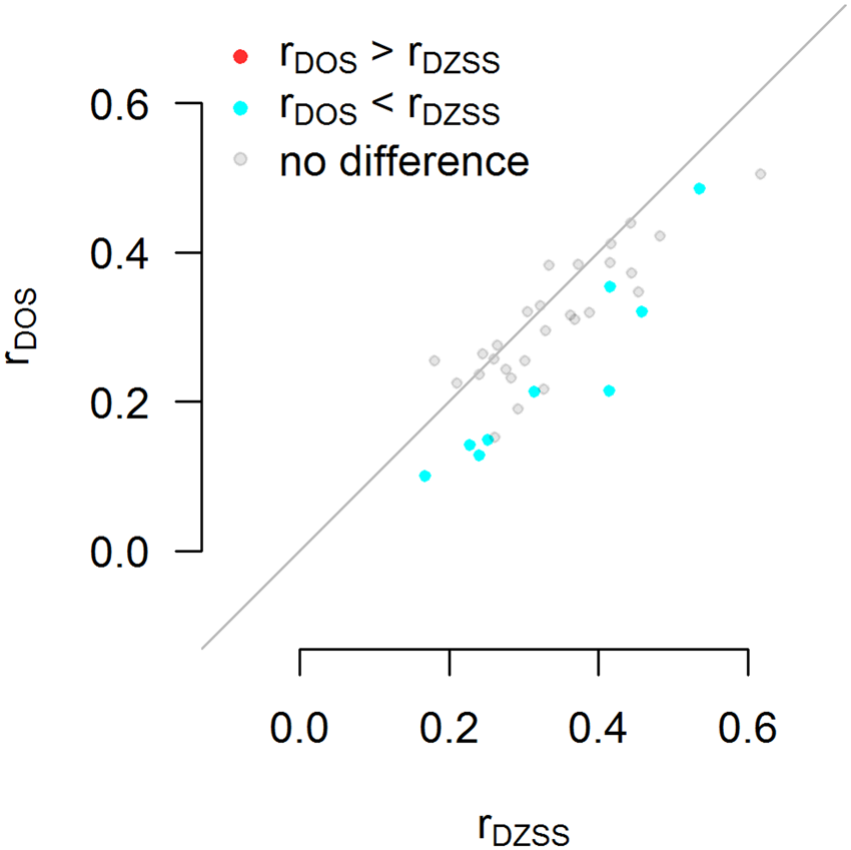
Nine trait categories (25%) show robust statistical evidence for reduced co-heritability between males and females. Scatterplot of all dizygotic (DZ) same-sex midpoint correlations (r_DZSS_ = 1/2 r_DZM_ + 1/2 r_DZF_) versus DZ opposite-sex correlations (r_DOS_) for a total of 36 trait categories. For each pair of it was tested whether r_DZSS_ = r_DOS_. Red dots indicate that r_DOS_ was significantly larger than r_DZSS_. Blue dots indicate that r_DZSS_ was larger than r_DOS_. Grey dots indicate no statistically significant difference after Bonferroni correction for 36 trait categories.

The contribution of sex-specific effects (r_DZSS_ = r_DOS_) could be tested in 36 trait categories revealing 9 categories (25%) with significant differences after Bonferroni correction, and 17 categories (47%) at the 0.05 significance level) (Fig. 4).

Finally, Table 2 shows the eight trait categories with robust statistical evidence for differences in genetic and/or shared environmental factors between males and females.

**Table 2:** Specific trait categories with evidence for different genes or environmental factors for males and females.

Trait	h^2^_diff_	p_h_^2^_diff_*	c^2^_diff_	p_c_^2^_diff_*	r_dzss-_r_dos_	p_dzss-dos_**
Food	0.03	1.00	−0.02	1.00	**0**.**10**	**4**.**2 × 10**^**−1**3^
Disorders of Puberty, Not Elsewhere Classified	0.24	0.18	−0.22	0.32	**0**.**20**	**4**.**6 × 10**^**−10**^
Eating Disorders	−0.08	1.00	0.01	1.00	**0**.**11**	**1**.**1 × 10**^**−6**^
Height	0.06	1.00	−0.04	1.00	**0**.**05**	**2**.**8 × 10**^**−4**^
Mental and Behavioural Disorders Due to Use of Tobacco	0.00	1.00	−0.08	1.00	**0**.**13**	**5**.**6 × 10**^**−4**^
Looking After One’s Health	0.01	1.00	−0.04	1.00	**0**.**10**	**1**.**4 × 10**^**−3**^
Specific Personality Disorders	−0.13	1.00	0.11	1.00	**0**.**09**	**4**.**9 × 10**^**−3**^
Weight Maintenance Functions	−0.04	1.00	0.02	1.00	**0**.**06**	**0**.**02**
Recurrent Depressive Disorder	0.08	1.00	−0.06	1	**0**.**07**	**0**.**04**
Functions of Brain	0.09	0.65	**−0**.**10**	**0**.**04**	0.03	0.09

h^2^_diff_ = (r_MZM_ - r_DZM_) − (r_MZF_ - r_DZF_); c^2^_diff_ = (2r_DZM_ - r_MZM_) − (2r_DZF_ - r_MZF_); r_dzss_ = midpoint between dizygotic male (r_DZM_) and female (r_DZF_) twin correlation and r_dos_ = dizygotic opposite-sex twin correlation. See Supplementary Dataset S1 for detailed information on all tested traits. *Bonferroni corrected p-value for 50 trait categories. **Bonferroni corrected p-value for 36 trait categories.

## Discussion

Although twin studies have reported sex-specific correlations for many traits, surprisingly few studies have systematically assessed the overall importance of sex-specific genetic and environmental influences. We present a comprehensive analysis of sex-specific genetic and environmental effects across all human traits that have been studied in twins to date, based on over two million twin pairs. Overall, we find little evidence for sex-related differences in the extent to which genetic or shared environmental influences contribute to the population variance. On the trait level only 1% of 2,608 traits showed robust statistical evidence for sex differences.

For some of the trait categories for which we did find sex-specific effects, differential sex effects can biologically be expected. For example, *Disorders of Puberty*, *Not Elsewhere Classified* includes traits like onset of puberty, which is measured differently in males and females (i.e. start of breast development in girls and change of voice in boys) and for which it is not unthinkable that different genes influence the specific bodily changes in boys and girls. Other traits, like *Height*, *Weight Maintenance Functions*, *Eating Disorders*, *Food*, *Specific Personality Disorders*, *Recurrent Depressive Disorder*, and *Looking After One’s Health* are measured similarly in males and females, but have been reported previously to be sexually dimorphic. For example, males and females have different fat percentages and fat distributions (*Weight Maintenance Functions*)^14^, different levels of physical activity and self-rated health (*Looking After One’s Health*)^15^. Although prevalence differences do not automatically imply etiological differences, it is noteworthy that food preferences, food intake (*Food*), and the prevalence of eating disorders are also different in males and females^16^, as well as in some personality disorders^17^. In addition, for all these trait categories the genetic contribution is much larger than that of shared environmental effects^10^. This suggests that sexual dimorphism for these categories is mainly driven by sex-specific genetic effects. In contrast, the categories *Mental and Behavioural Disorders Due to Use of Tobacco* and *Looking After One’s Health* show a relatively large contribution of shared environmental effects, suggesting that different environmental factors (as well as genetic factors) may contribute to the differences observed in males and females^10^.

Although our results show sex differences for some traits, it is yet unclear which genomic regions underlie these differences. Especially for sex dimorphic traits, sex differences may simply reflect differences on the sex chromosome.

Note that the heritability estimate (h^2^) and contribution of shared environmental factors (c^2^) are independent from the male-female genetic correlation and correlation due to shared environmental factors. While the former estimate how much genetic and shared environmental factors contribute in males and females, the latter estimates the extent to which the same factors contribute in males and females, irrespective of their magnitude.

Our results are in sharp contrast with the most prominent findings presented in a review by Gilks *et al*.^18^, which suggested that 50% of human traits have sex-specific heritabilities. An important difference between their review and our study is the number and type of traits. Gilks *et al*.^18^ analyzed 32 human traits that focused on anthropomorphic characteristics, where we analyzed a wide variety of over 2,600 traits. Furthermore, we based our analyses on over two millions of twin pairs, allowing robust assessment of sex-specific heritabilities in human traits. Of the 15 traits for which Gilks *et al*.^18^ reported sex differences in heritability we could confirm six sex-related etiological differences. These six traits were all part of the broader trait categories *Height*, *Weight Maintenance Functions*, and *Eating Disorders*.

The twin correlations used in this study have several levels of dependency. Many twin studies report on several, possibly related traits. Although this does not invalidate the individual p-values we report for example in Figs 1 and 2, it does imply that the Bonferroni correction will be somewhat conservative. The extent of this effect is difficult to assess, since it is not always known which correlations are (partly) based on the same sample.

Despite little evidence for sex differences in the relative contribution of genetic and shared environmental influences, one quarter of the investigated trait categories in our study do show evidence for differential genetic and/or environmental effects. Since many of these traits show small contributions of shared environment, it is likely that sex-specific genetic effects, as opposed to sex-specific environmental effects, play a role. Nonetheless, for a large majority of trait categories, including trait categories that are sexually dimorphic, we do not find evidence that the relative influences of genes and environment is different between males and females. This is consistent with recent studies based on SNP data that show large SNP-based genetic correlations between males and females across human traits^19–21^. For example, even the effect size of genetic variants related to sex-specific puberty-related traits like age at menarche and voice breaking show a relatively large correlation. These converging lines of evidence do not imply that sex-specific genetic variants do not exist for the majority of human traits. They do imply, however, that the overall contribution of sex-specific genetic variants to the genetic architecture is modest at best for most traits.

Although previous studies have performed similar analyses based on the classic twin model, the collection of phenotypes analyzed in this study is unprecedented by covering virtually all twin studies published to date, providing a comprehensive assessment of the sex differences across all human traits.

In conclusion, for the majority of traits, the relative influence of genes and environment is highly similar in males and females and genetic influences are typically not sex-specific. This suggests that for many traits the number of identified sex-specific genetic variants will be small compared to non-sex-specific variants. For those traits where we do report sexual dimorphism, sex-specific approaches may aid in future gene-finding efforts.

## Materials and Methods

We used the database of Polderman *et al*.^10^, which is a comprehensive database of virtually all twin studies published between 1958 and 2012. The database includes reported twin correlations from 2,748 twin studies for 17,804 traits classified into >300 official trait categories according to the official ICF^12^ and ICD-10^13^. In the current study we focus on traits as reported in the original study and trait categories classified according to the official ICF and ICD-10 sub-chapter classification. None of the trait variables have been adjusted for covariates before computing the twin correlations. However, since correlations are insensitive to sex differences in trait mean and variance, same-sex and opposite sex correlations in DZ twins are comparable for sex dimorphic traits as well.

Since only twin correlations are consistently reported in twin studies, we computed the commonly used least squares estimates of the heritability (h^2^ = 2(r_MZ_ − r_DZ_)) and relative contribution of shared environment (c^2^ = 2r_DZ_ − r_MZ_) for individual traits as well as the meta-analyzed trait categories^11^. A key assumption of the classic twin model on which the least squares estimates of h^2^ and c^2^ are based is that shared environmental influences are similar in monozygotic and dizygotic twin pairs respectively. In their comprehensive review of the twin literature, Polderman *et al*.^10^ only included twin studies in which siblings were brought up in the same nuclear family. Moreover, a previous study on several traits did not find significant differences in shared environmental factors between monozygotic and dizygotic twins^22^, showing at least for some human traits that this key assumption of the classic twin model is tenable.

We selected all traits for which sex-specific twin correlations and their standard errors (SEs) were available: the monozygotic male (r_MZM_) and female (r_MZF_), dizygotic male (r_DZM_) and female (r_DZF_), and opposite sex (r_DOS_) twin correlations. SEs were computed on the correlation scale $({\boldsymbol{se}}({\boldsymbol{r}})=\sqrt{\frac{1-{{\boldsymbol{r}}}^{2}}{{\boldsymbol{n}}-2}})$. From these twin correlations we derived the heritability (*h*^2^ = 2(r_MZ_ − r_DZ_); se(h^2^) = 4(se(r_MZ_)^2^ + se(r_DZ_)^2^)) and contribution of shared environment (c^*2*^ = 2r_DZ_ − r_MZ;_ se(c^2^) = 4se(r_DZ_)^2^ + se(r_MZ_)^2^) for males and females separately. We estimated the same-sex correlation by meta-analyzing the male and female dizygotic twin correlations using a fixed effects model after Fisher-z transformation. We used these sex-specific statistics to test the following three hypothesis: 1) male and female heritabilities are equal (h^2^_male_ = h^2^_female_), 2) male and female shared environmental effects are equal (c^2^_male_ = c^2^_female_), and 3) the opposite-sex twin correlation equals the DZ same-sex correlation (r_DZSS_ = r_DOS_). All hypotheses were tested with a Wald test (z = beta/se).

To account for similarity of traits within trait categories, we meta-analyzed all twin correlations within each trait category using a random effects model (see ref. 10 for details) and tested all three hypotheses for each trait category. We only included those traits for which at least 500 twin pairs were included in the meta-analysis and at least 10 studies contributed to the meta-analytic estimate. In addition, for each individual study at least 5 twin pairs needed to be present. This resulted in 50 trait categories (based on 2,608 individual trait statistics) with sufficiently reliable estimates to test our hypotheses (see Table 1). All three hypotheses were tested per trait as well as per trait category, based on the meta-analysis estimates of the relevant twin correlations. Bonferroni correction was applied per hypothesis for traits and trait categories respectively. See Supplementary Dataset S1 online for more elaborate descriptive statistics of the twin correlations and derived statistics.

Meta-analyzed data on the trait category level and R syntax used in the current study are included in this published article (and its Supplementary Information files). The raw data analysed and generated during the current study are available from the corresponding author on reasonable request.

## Electronic supplementary material

Supplementary Figures

Supplementary Dataset S1

Supplementary Code

## References

[1] Pan L, Ober C, Abney M (2007). Heritability estimation of sex-specific effects on human quantitative traits. Genet. Epidemiol..

[2] Ober C, Loisel DA, Gilad Y (2008). Sex-specific genetic architecture of human disease. Nat. Rev. Genet..

[3] Randall JC (2013). Sex-stratified Genome-wide Association Studies Including 270,000 Individuals Show Sexual Dimorphism in Genetic Loci for Anthropometric Traits. PLoS Genet.

[4] Dubois L (2012). Genetic and Environmental Contributions to Weight, Height, and BMI from Birth to 19 Years of Age: An International Study of Over 12,000 Twin Pairs. PLoS ONE.

[5] Rawlik K, Canela-Xandri O, Tenesa A (2016). Evidence for sex-specific genetic architectures across a spectrum of human complex traits. Genome Biol..

[6] Reinius B (2008). An Evolutionarily Conserved Sexual Signature in the Primate Brain. PLoS Genet.

[7] Rinn JL, Snyder M (2005). Sexual dimorphism in mammalian gene expression. Trends Genet..

[8] Yang X (2006). Tissue-specific expression and regulation of sexually dimorphic genes in mice. Genome Res..

[9] Wisniewski AB, Chernausek SD (2009). Gender in childhood obesity: Family environment, hormones, and genes. Gend. Med.

[10] Polderman TJC (2015). Meta-analysis of the heritability of human traits based on fifty years of twin studies. Nat. Genet..

[11] Falconer DS, Mackay TF, Frankham R (1996). Introduction to quantitative genetics (4th edn). Trends Genet..

[12] World Health Organization. *International classification of functioning*, *disability and health: ICF*. (World Health Organization, 2001).

[13] World Health Organization. International statistical classification of diseases and health related problems (The) ICD-10. (World Health Organization, 2004).

[14] Fried SK, Lee M-J, Karastergiou K (2015). Shaping fat distribution: New insights into the molecular determinants of depot‐and sex‐dependent adipose biology. Obesity.

[15] Azevedo MR (2007). Gender differences in leisure-time physical activity. Int. J. Public Health.

[16] Hudson JI, Hiripi E, Pope HG, Kessler RC (2007). The Prevalence and Correlates of Eating Disorders in the National Comorbidity Survey Replication. Biol. Psychiatry.

[17] Grant BF (2004). Prevalence, Correlates, and Disability of Personality Disorders in the United States: Results From the National Epidemiologic Survey on Alcohol and Related Conditions. J. Clin. Psychiatry.

[18] Gilks WP, Abbott JK, Morrow EH (2014). Sex differences in disease genetics: evidence, evolution, and detection. Trends Genet..

[19] Muñoz M (2016). Evaluating the contribution of genetics and familial shared environment to common disease using the UK Biobank. Nat. Genet..

[20] Day FR (2015). Shared genetic aetiology of puberty timing between sexes and with health-related outcomes. Nat. Commun..

[21] Yang J (2015). Genome-wide genetic homogeneity between sexes and populations for human height and body mass index. Hum. Mol. Genet..

[22] Derks EM, Dolan CV, Boomsma DI (2006). A test of the equal environment assumption (EEA) in multivariate twin studies. Twin Res. Hum. Genet. Off. J. Int. Soc. Twin Stud.

