# Author Correction: Majority of human traits do not show evidence for sex-specific genetic and environmental effects

**DOI:** 10.1038/s41598-018-36013-y

**Published:** 2018-12-21

**Authors:** Sven Stringer, Tinca J. C. Polderman, Danielle Posthuma

**Affiliations:** 1 Department of Complex Trait Genetics, VU University, Center for Neurogenomics and Cognitive Research, Amsterdam Neuroscience, Amsterdam, The Netherlands; 2 Department of Clinical Genetics, VU University Medical Centre, Amsterdam Neuroscience, Amsterdam, The Netherlands

Correction to: *Scientific Reports* 10.1038/s41598-017-09249-3, published online 17 August 2017

The original version of this Article contained a typographical error in the spelling of the author Tinca J.C. Polderman, which was incorrectly given as Tinca Polderman. This has now been corrected in the PDF and HTML versions of the Article.

