# Supplementary material for: Majority of human traits do not show evidence for sex-specific genetic and environmental effects
Source: Sci Rep. 2017 Aug 17;7:8688. doi: 10.1038/s41598-017-09249-3 (PMC5561094; doi:10.1038/s41598-017-09249-3)
Supplement: Supplementary file 1 — Supplementary Figures [file 41598_2017_9249_MOESM1_ESM.pdf]

Majority of human traits do not show evidence for sex-specific genetic and environmental effects

Sven Stringer, Tinca Polderman, and Danielle Posthuma

Supplementary Figures

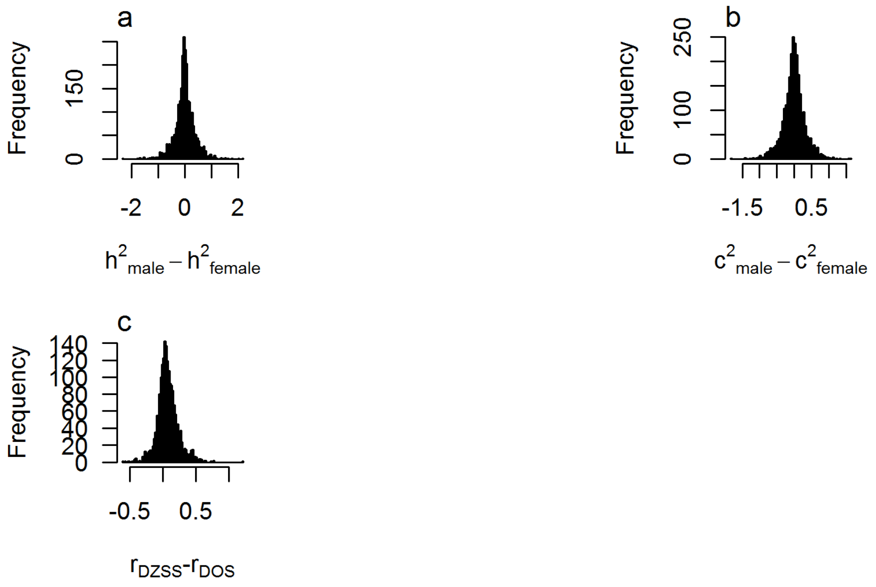

Figure S1. Simulated null distribution of sex-difference estimates across individual study traits based on reported standard errors assuming no sex-difference. Null distribution of sex-differences in (a) heritability estimate, (b) shared environmental effects, and (c) difference in same-sex and opposite sex correlation in dizygotic twin pairs.
